# Comparative Assessment of Free Energy Computational Methods for Revealing the Interactions Driving PARP1 Selective Inhibition

**DOI:** 10.1021/acs.jcim.6c00083

**Published:** 2026-04-18

**Authors:** Alejandro Feito, Natàlia DeMoya-Valenzuela, Cristian Privat, Andrés R. Tejedor, Marco DelValle-Carrillo, Sara Cembellín, Lucía Paniagua-Herranz, Adiran Garaizar, Javier Oller-Iscar, Alberto Ocana, Jorge R. Espinosa

**Affiliations:** † Department of Physical Chemistry, 16734 Universidad Complutense de Madrid, Av. Complutense s/n, Madrid 28040, Spain; ‡ Instituto Pluridisciplinar, Universidad Complutense de Madrid, P.° de Juan XXIII, 1, Moncloa - Aravaca, Madrid 28040, Spain; § Experimental Therapeutics Unit, Hospital Clínico San Carlos (HCSC), Instituto de Investigación Sanitaria San Carlos (IdISSC), Madrid 28040, Spain; ∥ Yusuf Hamied Department of Chemistry, 2152 University of Cambridge, Lensfield Road, Cambridge CB2 1EW, U.K.; ⊥ Department of Organic Chemistry, Universidad Complutense de Madrid, Av. Complutense s/n, Madrid 28040, Spain; # Data Science, 1569 Bayer AG, Alfred-Nobel-Straße 50, Monheim am Rhein 40789, Germany; ¶ PhAsIca Biosciences S.L, Calle Velázquez, 27, Madrid 28001, Spain

## Abstract

Accurate prediction of inhibitor selectivity across protein paralogues remains a central challenge in computational drug discovery. Here, we perform a comparative assessment of three computational methods—Molecular Mechanics/Poisson–Boltzmann Surface Area (MM/PBSA), Absolute Binding Free Energy (ABFE) and Umbrella Sampling (US) calculations—in their ability to recapitulate PARP1 versus PARP2 selectivity for eight clinically relevant PARP enzyme inhibitors used in ovarian, breast, and prostate tumors, among others. We demonstrate how MM/PBSA calculations offer rapid and qualitative insights but show pronounced sensitivity to the chosen static conformational pose, being particularly challenging for ligands with subtle energetic differences between distinct protein paralogues. In contrast, both ABFE and US calculations using atomistic models with explicit solvent result in substantially improved agreement with experimental binding affinities. The ABFE method exhibits the strongest quantitative correlation with experimental binding free energy differences, remarkably reproducing selectivity trends even among nearly isoenergetic complexes. Notably, our structural contact analysis reveals how contact connectivity controls ligand selectivity, providing valuable mechanistic and molecular insight into the key residues that stabilize each inhibitor in both protein enzymes. Together, our multimethod computational study contributes to elucidating potential chemical modifications across the ligand chemical space to enhance potency and specificity, informing the future design and evaluation of selective inhibitors for precision oncology, including therapies targeting homologous recombination-deficient cancers.

## Graphical abstract

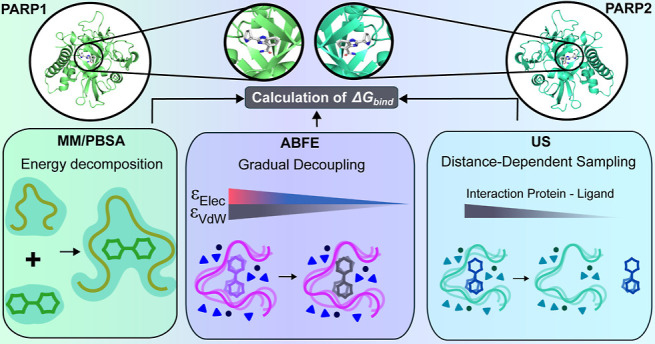

## Introduction

Poly­(ADP-ribose) polymerase 1 (PARP1) and 2 (PARP2) are key nuclear enzymes that serve as primary sensors of DNA single-strand breaks and orchestrators of the subsequent repair response.^1–3^ Both enzymes bind damaged DNA and catalyze the synthesis of poly­(ADP-ribose) chains that drive chromatin relaxation and recruit DNA repair factors.^4–7^ Targeting PARP1 and PARP2 with small-molecule inhibitors has become an effective therapeutic strategy,^8^ exploiting synthetic lethality in cancer cells with homologous recombination repair deficiency.^9,10^ Nevertheless, although PARP1 and PARP2 share overlapping biochemical functions and partially redundant activities,^2^ accumulating structural, biochemical, and genetic evidence demonstrates that PARP1 is the dominant mediator of DNA damage detection, whereas PARP2 plays a more specialized role in regulating chromatin organization and hematopoietic cell homeostasis.^2,10^ Disrupting PARP1 activity without substantially inhibiting PARP2 is therefore emerging as a powerful strategy for overcoming the hematologic toxicities associated with current PARP inhibitor (PARPi) therapy, in which off-target suppression of PARP2 significantly narrows the therapeutic window.^11–13^ This distinction has motivated a new generation of highly PARP1-selective inhibitors—exemplified by saruparib^14^ and NMS-P118 ^15^—designed to retain potent antitumor activity while minimizing dose-limiting cytopenias.^16^ Therefore, understanding and, more importantly, predicting the molecular determinants that generate such selectivity remains a central challenge in structure-guided computational drug discovery.^17^

Protein structure prediction combined with molecular docking provides plausible inhibitor binding modes and positional alignments within enzyme active sites; however, these static representations often overlook conformational plasticity, dynamic water networks, and entropic contributions that critically influence binding specificity.^18,19^ Computational free energy calculations can therefore play a central role in elucidating PARP1 versus PARP2 selectivity, with complementary strengths and limitations depending on the underlying physical approximations. Among the most widely used approaches, Molecular Mechanics/Poisson–Boltzmann Surface Area (MM/PBSA) calculations^20^ offer rapid end-point estimates of binding free energies based on molecular mechanics descriptions combined with implicit-solvent models.^21^ However, its accuracy partially depends on thorough conformational alignment and approximate entropic treatments. On the other hand, alchemical absolute binding free energy (ABFE) methods^22,23^ in combination with all-atom force fields enable chemically rigorous predictions by computing absolute binding free energies through thermodynamic transformations between ligand–protein and ligand-solvated states. A related approach, relative binding free energy (RBFE) calculations, estimates binding affinity differences by alchemically transforming one ligand into another, which can improve efficiency when applied to chemically similar ligand series.^24^ Recent developments have also explored hybrid strategies that combine ABFE and RBFE concepts to broaden their applicability and sampling efficiency.^25^ Nevertheless, despite their higher computational cost, the accuracy of these approaches can still be challenged by sampling barriers or slow protein rearrangements that accompany ligand binding in dynamic and flexible active sites. Finally, all-atom potential of mean force (PMF),^26,27^ usually implemented through umbrella sampling (US) or related enhanced-sampling techniques, resolves the free-energy landscape along physically meaningful dissociation coordinates, capturing both enthalpic and entropic contributions as the ligand traverses the conformational landscape of the catalytic cleft.^18^ While the binding free energy profile might moderately vary depending on the chosen association/dissociation pathway, the free energy minimum of the bound state is usually rather independent of such a pathway.^28^ Together, these computational methods, although entailing a certain degree of approximations, provide complementary insights into the thermodynamics and mechanisms of ligand binding and recognition, offering highly valuable insights into the binding affinity between potential drug candidates and protein binding pockets.

In this study, we perform a comprehensive assessment of eight clinically relevant PARP inhibitors—saruparib,^14^ NMS-P118,^15^ veliparib,^29,30^ olaparib,^12,30^ rucaparib,^31^ niraparib,^32^ talazoparib,^33^ and pamiparib. Using explicitly solvated atomistic molecular dynamics (MD) simulations,^34–38^ we characterize the conformational ensembles of each ligand–protein complex and evaluate binding free energies using MM/PBSA,^39^ alchemical ABFE,^40^ and US calculations.^41,42^ By directly comparing these three methodological approaches across a chemically diverse ligand set, we assess their predictive accuracy, computational performance, sensitivity to structural variability, and ability to capture the energetic determinants underlying protein selectivity. This integrative analysis reveals how subtle differences in the topology, dynamics, and solvation of the different PARP inhibitors translate into distinct binding modes and ultimate protein selectivity. Moreover, our structural contact analysis and energetic decomposition of the involved interactions reveal how contact connectivity controls ligand selectivity, providing mechanistic insight and a quantitative foundation for guiding the rational development of next-generation PARP1-selective therapeutics with improved efficacy and safety profiles.

### Computational Free Energy Methods

Binding energetics for all PARP1 and PARP2 complexes have been quantified using three complementary free energy methodologies—MM/PBSA, ABFE, and US calculations—each differing in their associated computational cost and methodological implementation. Their integration as a multitechnique computational platform provides a rigorous and mechanistically coherent thermodynamic description of ligand–protein selectivity. All simulations were initialized from experimentally resolved crystallographic structures of the corresponding ligand–protein complexes to ensure a consistent definition of the bound state across the different approaches. Subsequent system preparation and simulation parameters were then adapted according to the specific requirements of each methodology (e.g., alchemical λ scheme for ABFE or reaction-coordinate definition for US calculations) to ensure stable sampling and optimal convergence within each framework.

### Molecular Mechanics/Poisson–Boltzmann Surface Area (MM/PBSA) Calculations

In the MM/PBSA^50^ framework, the binding free energy is estimated from equilibrium ensembles using a molecular-mechanics description of both intramolecular and intermolecular interactions combined with an implicit treatment of solvation. For each representative configuration, the binding free energy is expressed as shown in eq 1.^51^

1 $$\begin{array}{ll} \Delta G_{bind} & =G_{complex}-\left(G_{protein}+G_{ligand}\right)\\ & =\Delta H+\Delta G_{solvation}-T\Delta S\\ & =\Delta E_{MM}+\Delta G_{Polar}+\Delta G_{NonPolar}-TS. \end{array}$$

Building on this formalism, the binding free energy is formally defined as the difference between the free energy of the protein–ligand complex and the sum of the free energies of the isolated protein and ligand G_complex_ – (*G*_protein_ + *G*_ligand_), which corresponds to the standard thermodynamic cycle underlying all binding free energy calculations. Here, G_complex_ denotes the free energy of the bound complex, *G*_protein_ is the free energy of the unbound protein, and *G*_ligand_ is the free energy of the unbound ligand. This formal definition can be applied to ensembles extracted from extensive MD simulations but can also be evaluated using a single representative conformation derived from experimental structures such as X-ray crystallography or cryo-EM.

This definition connects directly to a thermodynamic decomposition in which the free energy change upon binding can be expressed as the sum of an enthalpic term, a solvation contribution, and an entropic penalty, respectively (Δ*H* + Δ*G*_solvation_ – *T*Δ*S*), linking the macroscopic thermodynamic description of molecular recognition with the microscopic energetic and entropic processes involved in ligand binding. Here, Δ*H* corresponds to the enthalpic change in intramolecular and intermolecular interactions upon binding, Δ*G*_solvation_ accounts for desolvation effects, and *T*Δ*S* represents the entropic cost of constraining the relative motion and orientation of the binding molecule.

MM/PBSA provides a practical computational realization of this thermodynamic picture by rewriting these contributions in terms of quantities obtained from molecular mechanics and continuum solvation models: (i) the enthalpic component is approximated by the molecular mechanics energy (Δ*E*_MM_), which includes bonded, electrostatic, and van der Waals interactions; (ii) the solvation term is separated into a polar contribution (Δ*G*_Polar_), computed using either Poisson–Boltzmann (PB) or generalized Born (GB) implicit-solvent models in MM/GBSA^52,53^ calculations, and a nonpolar component (Δ*G*_NonPolar_), estimated from the solvent-accessible surface area (SASA) and associated with hydrophobic desolvation; and (iii) the entropic term (*S*), which accounts for the configurational entropy loss upon binding, typically evaluated through quasi-harmonic (QHA) or normal-mode analysis (NMODE).

While both the Poisson–Boltzmann (PB) and the generalized-Born (GB) approaches seek to approximate the electrostatic contribution to solvation, they differ fundamentally in mathematical rigor and computational cost. The PB model solves the Poisson–Boltzmann equation numerically as shown in eq 2.^54^

2 $$\nabla \cdot \left(\epsilon \left(r\right)\nabla \phi \left(r\right)\right)-\sum_{i}c_{i}^{0}z_{i}e\exp\left(-\frac{z_{i}e\phi \left(r\right)}{k_{B}T}\right)=-\rho \left(r\right)$$

where it describes how the electrostatic potential ϕ­(**r**) is distributed around a biomolecule in an ionic solution. In this framework, ϵ­(**r**) denotes the spatially dependent dielectric constant, which takes low values inside the protein cavity and high values outside the solvent. The term ρ­(**r**) represents the fixed charge density of the solute, typically arising from atomic partial charges. The summation term compensates for the contribution of mobile ions in solution, where *c*_*i*_^0^ represents the bulk concentration of ionic species *i*, *z*_*i*_ is their valence, and *e* is the elementary charge. The exponential factor reflects the Boltzmann distribution of ions in response to the local electrostatic potential, with *k*_B_ being the Boltzmann constant and *T* being the temperature. In the linearized form of the equation, the ionic response is approximated by the Debye–Hückel length, κ, which encodes the screening effect of electrolytes in solution. Altogether, the Poisson–Boltzmann equation provides a continuum description of electrostatic interactions by coupling the fixed charges of the solute with the redistributable charges of the surrounding ionic environment, providing a more accurate continuum description of the electrostatic environment at the cost of a significantly higher computational demand. However, despite its success, the Poisson–Boltzmann framework presents important limitations due to the absence of an explicit molecular description of the solvent. First, desolvation barriers are only implicitly treated, preventing an accurate description of the free-energy cost associated with removing structured water molecules from binding interfaces.^20^ Second, the continuum approximation neglects the discrete and directional nature of solvent molecules,^50^ thus missing explicit hydrogen-bond networks that can stabilize specific configurations. Additionally, the model does not capture solvent-mediated interactions and fluctuations, including hydrophobic effects and entropic contributions arising from water reorganization.^50^ Finally, the use of a spatially averaged dielectric constant neglects local dielectric heterogeneity and polarization effects, particularly relevant in confined or heterogeneous environments such as binding pockets.^20^

In contrast, GB models—including the modern GB5 ^55^ variant—approximate the PB solution analytically through pairwise descreening functions, achieving a substantial reduction in computational cost while maintaining reasonable accuracy for most biomolecular systems. GB5 in particular improves the effective Born radius calculation and dielectric screening terms, yielding results that more closely approach PB-level accuracy but still fall short in highly charged or topologically complex environments. In this study, we employed the widely used PB and GB models to perform a consistent comparative assessment. Although more recent variants have been recently proposed,^56^ the present work focuses on these established implementations to ensure methodological consistency across the methods evaluated. In practice, PB is preferred when accuracy is critical,^57^ whereas GB5 offers a practical balance between speed and precision for large-scale or high-throughput MM/GBSA calculations. In the Supporting Information, we provide further details of these two methodologies.

### Alchemical Absolute Binding Free Energy (ABFE) Calculations

To obtain chemically accurate estimates of protein–ligand binding affinities, we perform atomistic alchemical ABFE simulations in which we gradually transform ligand *A* into ligand *B* using a coupling parameter λ that interpolates between the two Hamiltonians. The associated free energy change is computed using the Zwanzig relation as shown in eq 3.^58^

3 $$\Delta G_{A\to B}=-k_{B}T\;\ln{\left\langle \exp\left[-\beta \left(U_{B}-U_{A}\right)\right]\right\rangle }_{A}$$

where *U*_*A*_ and *U*_*B*_ refer to the potential energies of the system under the Hamiltonians of states *A* and *B*, respectively; *k*_B_ is Boltzmann’s constant; *T* is the absolute temperature; $\beta ={\left(k_{B}T\right)}^{-1}$ ; and ⟨···⟩_*A*_ denotes an ensemble average over configurations sampled in both states *A* and *B*. This relation provides a rigorous statistical-mechanical framework to compute free energy differences between two chemical states from an ensemble of microscopic configurations.

To ensure thermodynamic consistency, positional restraints must be applied during the alchemical transformations. Harmonic constraints prevent translation of the ligand within the binding pocket, while additional restraints suppress global rotation and translation of the protein. Specifically, ligand translation needs to be restricted when varying the Hamiltonian by applying positional restraints to a single heavy atom, minimizing perturbation to the rest of the molecule. In the protein, four strategically selected residues are constrained to suppress the overall translation and rotation. This approach preserves a well-defined relative frame of reference between the protein and ligand while allowing the system sufficient structural flexibility to undergo conformational changes. Such restraints ensure stable overlap of conformational ensembles across intermediate λ states connecting both Hamiltonians, *A* and *B*, where molecular interactions are partially switched off, and positional alignment cannot be maintained through the inherent intermolecular interactions of the system.

In the alchemical route, the gradual decoupling of the ligand from its environment follows the standard two-stage scheme employed in absolute binding free energy calculations.^59^ First, electrostatic interactions are turned off smoothly as λ increases, thereby neutralizing the ligand without introducing large steric perturbations. Once the charges have been fully removed, van der Waals interactions are gradually scaled down using soft-core potentials that prevent numerical instabilities of atoms from losing their excluded-volume repulsion. This sequential treatment—electrostatics first, van der Waals second—ensures a physically meaningful pathway with improved configuration overlap between neighboring windows.^60,61^ All alchemical transformations were performed using independent equilibrium simulations at discrete λ values (fixed-λ windows) rather than nonequilibrium switching trajectories. Free energy differences were subsequently estimated from these equilibrated states using standard equilibrium estimators (Figure S9 in Supporting Information for the overlap matrix of saruparib bound to PARP1 and PARP2).

Free energy differences along the full λ schedule are subsequently combined using the multistate Bennett acceptance ratio (MBAR),^62^ which provides a statistically optimal estimator of Δ*G* by explicitly exploiting the overlap of configurational ensembles between adjacent λ-windows. In essence, the MBAR generalizes the Bennett acceptance ratio and can be expressed as shown in eq 4.^62^

4 $${\mathrm{f̂}}_{k}=-\ln\sum_{n=1}^{N}\frac{\exp\left[-\beta U_{k}\left(x_{n}\right)\right]}{\sum_{j=1}^{K}N_{j}\;\exp\left[{\mathrm{f̂}}_{j}-\beta U_{j}\left(x_{n}\right)\right]}$$

where ${\mathrm{f̂}}_{k}$ accounts for the dimensionless free energy of state *k*, *U*_*k*_(*x*_*n*_) is the potential energy of configuration *x*_*n*_ evaluated in the state *k*, *N*_*j*_ is the number of samples collected in state *j*, and the sum runs over all collected configurations across *K* states. This formulation effectively performs an overlap sampling^63^ by reweighting contributions from all windows according to how well each configuration represents the neighboring states, ensuring robust and statistically efficient estimates of Δ*G*. Relative binding free energies, Δ*G*_bind_, can be finally obtained by subtracting the corresponding alchemical transformation in aqueous solution, thereby connecting the free energy difference in the bound state with that in the unbound solvated state. In this way, eq 3 represents the formal statistical-mechanical definition of the alchemical free energy change, while the λ-dependent simulations and the corresponding MBAR analysis provide the practical computational implementation used to compute binding free energies from molecular simulations. Please see Section SII–III of the Supporting Information for further details of the system preparation and method implementation in this work, respectively. Several recent developments in alchemical free-energy calculations combine FEP with enhanced sampling strategies, such as REST2 or Hamiltonian replica-exchange methods, to improve the exploration of slow degrees of freedom, including protein side-chain rearrangements, water-network reorganization, and binding-site flexibility.^64–66^ These approaches can accelerate convergence and increase robustness for systems in which large conformational changes occur. However, in the present study, we employed a conventional FEP implementation since the ligands, due to their molecular size and structure, are unlikely to undergo major conformational rearrangements within the binding pocket into a different pose or binding mode.

### Umbrella Sampling (US) Calculations

Finally, to complement both the end-point and alchemical ABFE calculations, US simulations are employed to resolve the free energy landscape of ligand binding association along a physically meaningful reaction coordinate. The ligand was incrementally displaced from the catalytic binding site of PARP1 (or PARP2) to the bulk solution, respectively, while applying harmonic biasing potentials in each umbrella window^41^ as shown in eq 5

5 $$U_{i}\left(z\right)=\frac{1}{2}k_{i}{\left(z-z_{i}\right)}^{2}$$

where *z* represents the ligand–protein separation reaction coordinate, *z*_*i*_ is the window center, and *k*_*i*_ is the force constant. Because PMF reconstruction is sensitive to global rigid-body motions, both the protein and the ligand are subjected to partial rotational and translational restraints to ensure a stable reference frame throughout the dissociation/association pathway. In the ligand, these restraints are applied to approximately six heavy atoms, limiting the overall translation while allowing internal flexibility and partial conformational rearrangements. In the protein, four selected residues are restrained to suppress global rotation and translation. These constraints prevent spurious drift and ensure that the reaction coordinate reflects genuine unbinding events rather than system-level displacements while still allowing the system to explore relevant conformational changes.

The unbiased potential of mean force *A*(s) is calculated by combining the probability distributions from all umbrella sampling windows and correcting for the biasing potential *w*(s) applied in each window, which can be expressed as shown in eq 6.^67^

6 $$A\left(s\right)=A^{\prime }\left(s\right)-w\left(s\right)-k_{B}T\;\ln\left\langle \exp\left[\frac{-w\left(s\right)}{k_{B}T}\right]\right\rangle$$

where *A*′(s) is the PMF from the biased simulation, *w*(s) is the harmonic biasing potential, *k*_B_ is the Boltzmann constant, *T* is the temperature, and the angle brackets denote an average over the simulation windows. The final, unbiased PMF *W*(*z*) is then reconstructed from all windows using the weighted histogram analysis method (WHAM), yielding a continuous free energy profile that resolves the interplay of enthalpic interactions, conformational flexibility, hydration, and entropic penalties associated with confinement in the catalytic binding pocket. After the application of standard-state corrections, the depth of the PMF yields an absolute estimate of the binding free energy (Δ*G*_bind_). Please see Section SII–IV of the Supporting Information for further details of the system preparation and implemented methodology in this work, respectively. The unbinding of the ligand was monitored using a reaction coordinate defined by the center-of-mass (COM) distance between the ligand and the protein binding pocket. This coordinate was selected because it represents the pathway with minimal steric hindrance in the three-dimensional structure and was applied uniformly across all systems (see Figure S10 in the Supporting Information to see the chosen reaction coordinate across the ligand, i.e., veliparib with PARP1)

Together, these three complementary methodological layers—MM/PBSA evaluation, alchemical ABFE transformations analyzed with MBAR, and PMF-based reconstruction across the dissociation pathway with WHAM—provide a coherent, complementary, and quantitatively grounded framework for dissecting the molecular origins of selective PARP1 inhibition. Through this multistep methodology, we evaluate the relative binding free energy differences (ΔΔ*G*_bind_) between PARP1 and PARP2 for each ligand, enabling a systematic assessment of ligand selectivity across these homologous protein targets.

In Figure 1, we provide a schematic overview of the computational strategies employed to estimate protein–ligand binding free energies. The workflow begins with the preparation of the initial protein–ligand complex configuration, which is subjected to atomistic molecular dynamics simulations in explicit solvent, including water molecules and ions. Using GROMACS (version 2023),^68^ all simulations are performed with the atomistic a99SB-*disp* force field^69^ for the protein–protein interactions, the TIP4P-*disp* water model, and ligand parameters derived from the OpenFF^70^ toolkit, ensuring a consistent and accurate description of protein, ligand, and water/ion interactions. Both US and ABFE calculations are performed using this force field. In contrast, MM/PBSA can be performed directly on the initial structures experimentally resolved—the following PDB codes are used in this study: PARP1:9ETQ,^14^ 7KK4,^30^ 7KK6,^30^ 5A00,^15^ 7KK5,^30^ 6VKK,^71^ 7CMW,^49^ 7KK3;^30^ PARP2:4TVJ,^30^ 3KJD,^29^ 4ZZY,^15^ 8HLQ,^72^ 8HKO,^72^ 8HKS,^72^ and 4PJV^73^—providing a computationally quick end-point estimation of binding affinity. MM/PBSA decomposes the total energy into molecular mechanics and solvation contributions using the Poisson–Boltzmann equation for polar solvation. FEP employs a gradual alchemical decoupling of the ligand, separating van der Waals and electrostatic interactions across a series of λ-windows, with the resulting free energy differences combined via multistate estimators such as MBAR, capturing subtle enthalpic, entropic, and solvation effects with minimal positional restraints. Finally, US simulations compute the free-energy profile along a predefined reaction coordinate, typically by pulling the ligand out of the binding pocket with controlled positional restraints, thereby characterizing the energetic barriers and intermediate states along the unbinding pathway. The schematic from Figure 1 also highlights the relative computational cost of the three methods, with MM/PBSA being the fastest, followed by ABFE and US, and shows how all approaches ultimately converge on the calculation of the binding free energy, Δ*G*_bind_, enabling a comparative assessment of ligand binding affinities and selectivity across homologous protein targets.

**Figure 1:**
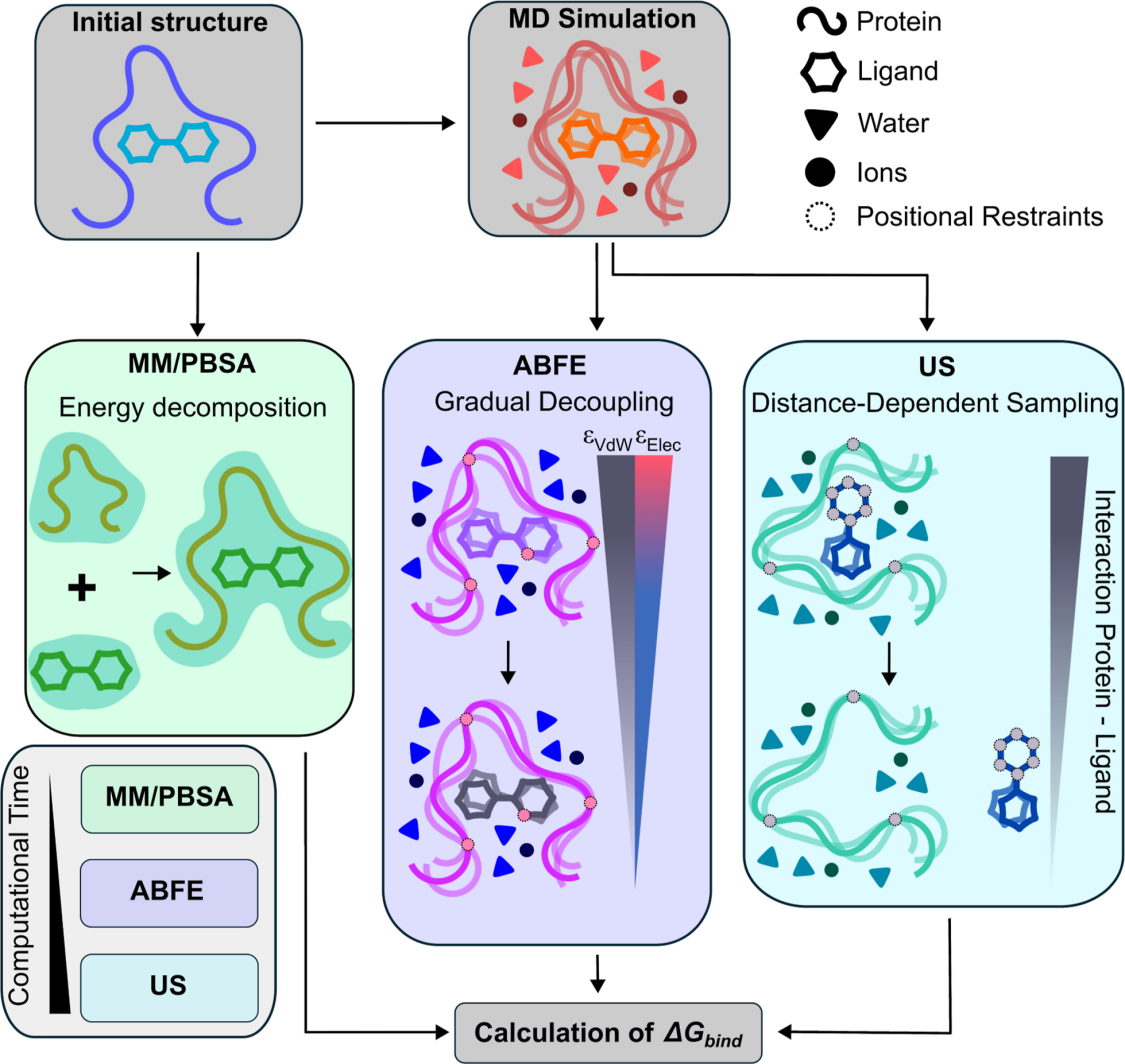
Schematic representation of the three computational approaches used in this work to estimate the binding free energy (Δ*G*_*bind*_) of a given ligand in different protein paralogues. The relative binding affinity can be evaluated by MM/PBSA, which estimates the free energy through energy decomposition of representative conformations; Absolute Binding Free Energy (ABFE), where interactions between the ligand and its environment are gradually decoupled; and Umbrella Sampling (US), which quantifies the free energy profile as a function of the protein–ligand dissociation pathway. The relative computational cost of each method is also included.

In terms of statistical uncertainty, the associated error for both US and ABFE calculations is estimated using a block-bootstrap^74^ analysis applied to extensive MD equilibrium trajectories. For the all-atom US simulations, each system was simulated for a cumulative total time of 1000 ns, from which the first 400 ns are discarded as equilibration time and the remaining 600 ns are used for production. The production trajectory is divided into multiple subwindows that effectively correspond to five statistically independent replicas across each Umbrella Sampling window. This approach is statistically equivalent to performing several shorter independent simulations for an ergodic system at thermodynamic equilibrium while avoiding additional relaxation times associated with identical initial conditions. An analogous procedure was applied to the all-atom ABFE calculations, which accumulated a total simulation time of 75 ns per system. The extended trajectory is treated as a sequence of approximately four to five independent replicas, and statistical errors are quantified using the bootstrap methods implemented in GROMACS.

## Results and Discussion

### Computational Assessment of PARP1 vs PARP2 Inhibitor Selectivity

Comparative assessment of computational methods for predicting PARP inhibitor selectivity is essential for determining the reliability of free energy calculations that can guide the design of more potent inhibitors in drug discovery. This is particularly relevant for protein families such as PARPs, whose members share highly similar dimensional structures but differ in sequence so that subtle energetic differences ultimately determine selectivity. Our goal in this section is therefore 2-fold: first, to examine how the predicted PARP1/PARP2 selectivity evolves as increasingly rigorous and time-demanding computational methodologies are applied; and second, to assess whether these distinct approaches—MM/PBSA, ABFE, and US—converge on consistent qualitative trends across a chemically diverse set of inhibitors. To this end, we first focus on four representative ligands: saruparib and NMS-P118, which are well characterized as highly selective PARP1 inhibitors,^14,15^ and veliparib and olaparib, which are known to exhibit broader, nonselective activity within the PARP protein family.^29,30^ All simulations are performed exclusively on the catalytic binding site of each protein with the ligands, see Section SIV of the Supporting Information for further details of the PDB structures and PARP1/2 active site sequence. By including ligands that span these two extremes of selectivity, we ensure that the comparative analysis challenges each computational method not only with subtle energetic differences but also with cases where substantial shifts in binding preference take place. This framework allows us to evaluate not only the numerical accuracy of each technique but also the stability and robustness of its relative predictions as simulation complexity increases. Importantly, we note that the size of the ligand set considered in this work reflects the constraints of the PARP1/PARP2 selectivity landscape. Structural and biochemical data are imbalanced, with approximately 70 ligand-bound PARP1 crystal structures available in the PDB compared to only about 10 for PARP2. Moreover, only a small subset of inhibitors (<10) have directly comparable IC_50_ values to both protein paralogues for benchmarking our simulations. To ensure structural consistency and minimize model-dependent uncertainties, all complexes were initialized from experimentally resolved crystallographic structures available in the PDB. Table 1 summarizes the ligands used in this study together with the corresponding experimental IC_50_ values.

**Table 1:**
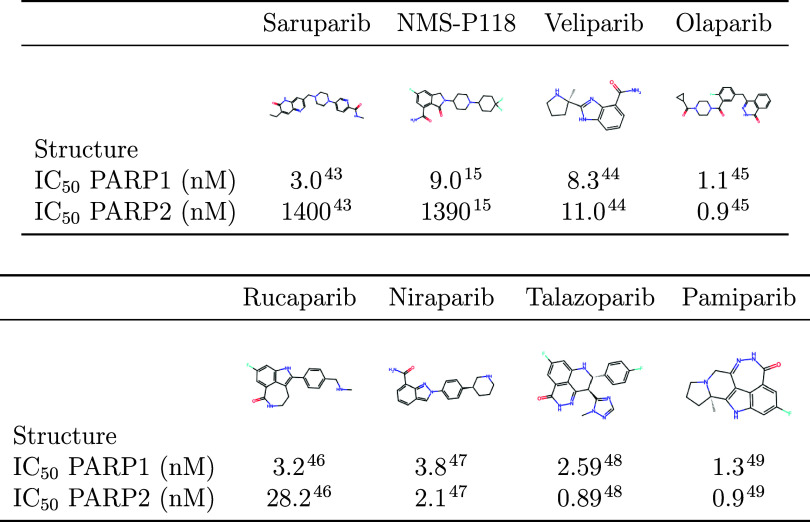
Structures and IC_50_ Values for PARP Inhibitors Used in This Study

To establish the context for this comparison, Figure 2A shows the approximate CPU times required to perform these calculations on a single CPU core with these three different methodologies. A striking feature of this panel is the difference in scale: MM/PBSA differs by several orders of magnitude in comparison with ABFE and US methods. Such a major difference arises naturally from the distinct level of approximations on which each method is built. MM/PBSA relies primarily on static energetic evaluations with an implicit solvent representation, whereas ABFE and US achieve greater thermodynamic rigor through explicit sampling of protein–ligand interactions and explicit solvent dynamics. As a result, direct comparison of binding free energies across methods is neither meaningful nor expected; instead, the emphasis must be placed on relative patterns between distinct inhibitor binding free energies. The selectivity is thus quantified through the relative binding free energy difference, ΔΔ*G*_bind_, defined as shown in eq 7

7 $$\Delta {\left(\Delta G\right)}_{PARP1-PARP2}=\Delta G_{bind}^{PARP1}-\Delta G_{bind}^{PARP2}$$

where Δ*G*_bind_^PARP1^ and Δ*G*_bind_^PARP2^ denote the binding free energy of the same ligand to PARP1 and PARP2, respectively.

**Figure 2:**
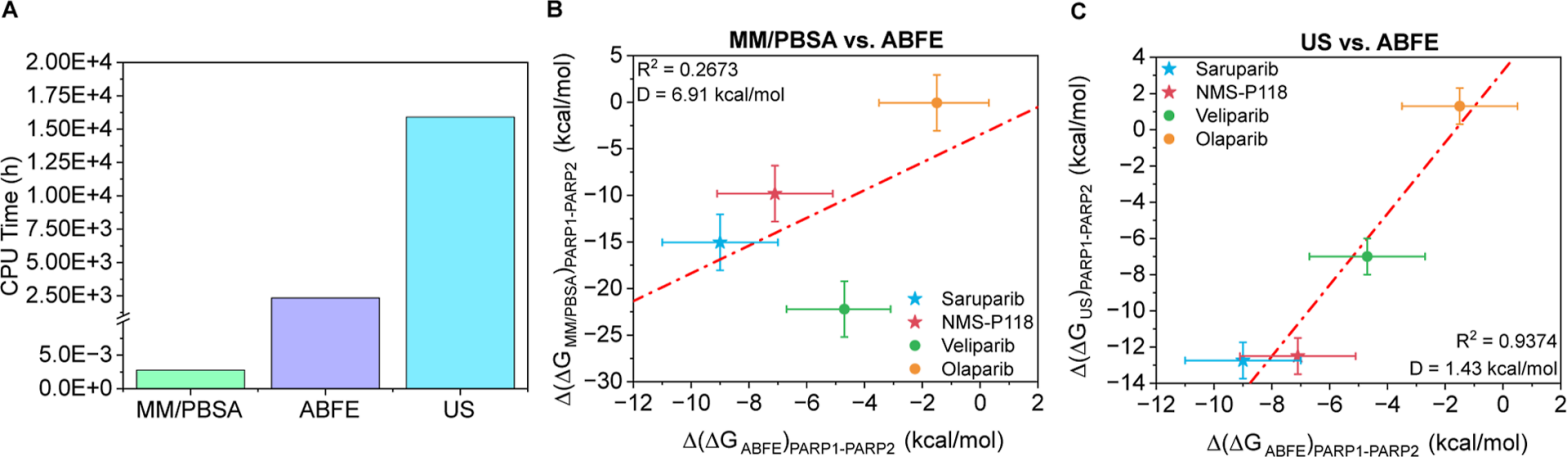
(A) Required simulation time (in hours) of a single CPU core for performing the binding energy prediction using the different tested methods. Comparison of the difference (Δ) of binding free energy (Δ*G*) between PARP1 and PARP2 (Δ­(Δ*G*)_PARP1–PARP2_) for four inhibitors: saruparib (blue star), NMS-P118 (red star), veliparib (green circle), and olaparib (yellow circle) using different computational approaches. (B) Correlation between MM/PBSA and ABFE results. (C) Correlation between US and ABFE calculations. Error bars represent the standard deviation of the simulations. Specific inhibitors of PARP1 are plotted as stars and nonspecific inhibitors as circles. The red dashed line depicts the linear regression of the data shown.

This perspective becomes clearer in Figure 2B, where we initially compare the MM/PBSA and FEP predictions. Despite their methodological differences, the two approaches reveal a consistent linear trend for three of the studied inhibitors: saruparib, NMS-P118, and olaparib. The correlation for these compounds indicates that, although the absolute values differ substantially between MM/PBSA and ABFE, both methods capture similar relative selectivity relationships. However, the overall coefficient of determination is modest with *R* ^2^ = 0.2673, reflecting that the linear agreement is limited when considering all four ligands together. Veliparib is chiefly responsible for this reduction in the correlation, as it does not follow the trend defined by the other inhibitors and appears as a clear offset. This deviation strongly suggests that MM/PBSA cannot adequately account for subtle structural and solvation features of veliparib with respect to the rest of the ligands, which are better represented in atomistic ABFE simulations. The issue likely stems not from an intrinsic anomaly of the ligand but rather from limitations of the MM/PBSA protocol—particularly its sensitivity to subtle conformational rearrangements of the molecule within the binding site and solvent-mediated intermolecular interactions. Veliparib thus serves as a diagnostic case illustrating where increased sampling rigor and detailed atomistic interactions begin to substantially influence the predicted selectivity.

To quantify how much MM/PBSA versus ABFE predictions deviate from an ideal perfect linear relationship, we compute a deviation metric *D*, defined as shown in eq 8

8 $$D^{2}=\frac{1}{n}\sum_{i=1}^{n}{\left(y_{fit,i}-y_{data,i}\right)}^{2}$$

where *n* is the number of data points (ligands), *y*_fit,*i*_ is the value on the regression line (the ideal fitted value), and *y*_data,*i*_ is the actual computed simulation value. In essence, *D* measures the mean squared deviation of the data from the ideal linear fit, where smaller *D* values indicate that the points cluster more tightly around the correlation line.

In our comparison of MM/PBSA versus ABFE calculations, we obtain *D* = 6.91 kcal/mol, reflecting a considerable deviation of veliparib’s prediction around the fitted line. In the context of the intrinsic uncertainties of each method, MM/PBSA itself is known to exhibit relatively large errors, often around 2–3 kcal/mol,^75^ while ABFE is generally slightly more precise, with typical uncertainties of 1–2 kcal/mol.^76^ Therefore, part of the scatter and the modest *R* ^2^ value observed in Figure 2B can be attributed to the intrinsic limitations of both methods rather than to their associated statistical uncertainty.

In Figure 2C, we compare ABFE and US calculations, two advanced methodologies that entail all-atom MD simulations with an explicit solvent. Here, the agreement is markedly stronger: all four inhibitors lie close to a linear trend with a high coefficient of determination *R* ^2^ = 0.9374. This strong correlation suggests that ABFE and US largely converge on a consistent description of PARP1 versus PARP2 selectivity for the considered PARP inhibitors. For the ABFE versus US comparison, the deviation metric is *D* = 1.43 kcal/mol, indicating a much closer alignment between these two methods than between MM/PBSA and ABFE. The small *D* is consistent with the typical uncertainties of both methods, where US calculations usually entail statistical uncertainties of ∼1 kcal/mol.^77^ Thus, the observed spread is comparable to the intrinsic error of the methods, and most of the residual scatter likely reflects statistical fluctuations rather than systematic disagreement.

Although ABFE and US simulations show strong overall agreement, the two approaches moderately differ in the level of discrimination they provide among the considered inhibitors. In particular, while ABFE captures noticeable differences across all four compounds, the PMF profiles for the two selective inhibitors (see Figure SI in the Supporting Information) yielded nearly identical free energy values. This behavior reflects several methodological limitations that affect each technique in distinct ways. Two important limitations of ABFE are directly related to the statistical efficiency of alchemical sampling. First, ABFE requires sufficient phase-space overlap between adjacent λ-windows. When the configurational ensembles at successive λ values become too dissimilar, the overlap of the energy distributions deteriorates, leading to large variances in the exponential averaging estimator and poor simulation convergence. This phenomenon has been well documented as a major source of noise and instability in ABFE calculations.^78^ Second, there is a nontrivial interplay between simulation time and statistical efficiency. Although longer simulations per window can, in principle, improve sampling, they may also exacerbate divergence between windows if slow conformational rearrangements occur during alchemical transformation. As a result, excessive per-window simulation time can counterintuitively reduce phase-space overlap and decrease the accuracy of the free energy estimates. This issue has been highlighted in recent methodological analyses, which recommend using multiple independent windows with moderate sampling rather than excessively long trajectories in each window.^79,80^ For these reasons, in our study, the ABFE protocol is constructed using the same number of λ-windows for all four inhibitor-protein systems and the same number of restraints in each system. This uniform setup ensures that any differences in the resulting free energy estimates arise from the physical behavior of the atomistic force field rather than from methodological inconsistencies in window spacing or statistical sampling. By standardizing the alchemical resolution across all transformations, we minimize the possibility that variations in phase-space overlap or sampling quality can introduce artificial discrepancies in the comparison between different PARP inhibitors.

In contrast, US calculations intrinsically rely on a predefined reaction coordinate^81,82^ along which the ligand is pulled out of the binding pocket. The resulting free energy profile is therefore sensitive to the choice of the coordinate: if it does not reflect the physically relevant unbinding pathway or if multiple pathways exist but are not sampled, the US may underestimate or oversmooth energetic differences between ligands. Moreover, US calculations typically require positional restraints on the ligand to maintain a controlled pulling trajectory (see Section SIV for further methodological details on the US calculations and obtained PMF profiles). These restraints can hinder molecular rearrangements and lead to artificially overestimated free energy barriers across the dissociation pathway.^83^ For this reason, in our study, the same reaction coordinate and the same number of restrictions are used for all four inhibitors, ensuring that differences between PMF profiles do not arise from arbitrary choices across the pulling path. Nevertheless, it must be acknowledged that any fixed reaction coordinate implicitly favors certain unbinding pathways over others and may therefore advantage some ligand–protein complexes relative to others in an indirect, subtle manner, which is extremely difficult to quantify.

Altogether, these considerations reveal a coherent progression: while MM/PBSA can provide a rapid, approximate qualitative picture, the more rigorous ABFE and US methods progressively reduce uncertainty, converge toward self-consistent values, and align more closely with each other within their intrinsic error margins. This underscores both the utility and limitations of each approach and highlights the importance of rigorous methods when quantitative accuracy is required in binding free energy predictions.

### Comparative Assessment of Computational Binding Free Energies through Different Methods against Experimental Values

To directly evaluate the ability of the all-atom amber99sb-*disp* force field—using both ABFE and PMF calculations—to reproduce the experimentally reported selectivity for different PARP inhibitors, we now compare the simulation-derived free energy differences to the experimental binding affinities reported for PARP1 and PARP2. Experimental IC_50_ values (i.e., the concentration of the ligand required to reduce the activity of the target protein by 50%) are converted into binding free energies according to the following relationship, as shown in eq 9

9 $$\Delta G_{binding}=RT\;\ln\left({IC}_{50}\left(M\right)\right)$$

where *R* is the gas constant and *T* = 300 K. Experimental IC_50_ values are taken as follows: saruparib, 3 nM for PARP1 and 1400 nM for PARP2;^43^ NMS-P118, 9 nM for PARP1 and 1390 nM for PARP2;^15^ veliparib, 8.3 nM for PARP1 and 11 nM for PARP2;^44^ and olaparib, 1.1 nM for PARP1 and 0.9 nM for PARP2,^45^ and then converted into binding Δ*G* values (see Table 1 for the experimental IC_50_ values used in this study, along with the chemical structures of the different PARP inhibitors). The associated uncertainty in the experimental binding free energies is estimated by propagating the standard deviation (σ) of the IC_50_ values reported across different assays in the ChEMBL database, providing an experimental error bar that reflects the variability inherent to heterogeneous biochemical measurements using the expression as shown in eq 10

10 $$\sigma_{\Delta G_{binding}}\approx RT\frac{\sigma_{{IC}_{50}}}{{IC}_{50}}$$

In Figure 3A, we present the comparison between experimentally derived differences in binding free energies between PARP1 and PARP2 (Δ­(Δ*G*)) and those obtained from MM/PBSA calculations. Consistent with the observations made earlier for the MM/PBSA vs ABFE comparison, the overall agreement with experiment is weak: the correlation coefficient is low (*R* ^2^ = 0.1269), and the deviation from an ideal linear relationship is significantly high (*D* = 7.54 kcal/mol, eq 8). A closer inspection reveals that the poor global correlation is primarily driven by veliparib, which appears as a clear outlier and does not follow the trend defined by the three other ligands. In contrast, the remaining three inhibitors—saruparib, NMS-P118, and olaparib—exhibit a more coherent linear trend between experimental and MM/PBSA-derived selectivities, mirroring the behavior observed in Figure 2B. This pattern reinforces that MM/PBSA struggles to capture subtle selectivity differences in highly similar ligand–protein complexes. A potential source of these deviations lies in the approximations inherent to the implicit solvent treatment employed in both the PBSA and GBSA calculations. In these approaches, solvent effects are represented through continuum electrostatic models, which approximate specific water-mediated ion interactions and hydrogen-bonding networks within the binding pocket. In systems such as PARP1 and PARP2, where ligand recognition often involves structured water molecules and subtle rearrangements of local interaction networks, these effects may play a significant role in determining the global binding free energy. Consequently, the simplified solvent representation used by the standard MM/PBSA method can lead to inaccuracies when small energetic differences between closely related complexes must be resolved.

**Figure 3:**
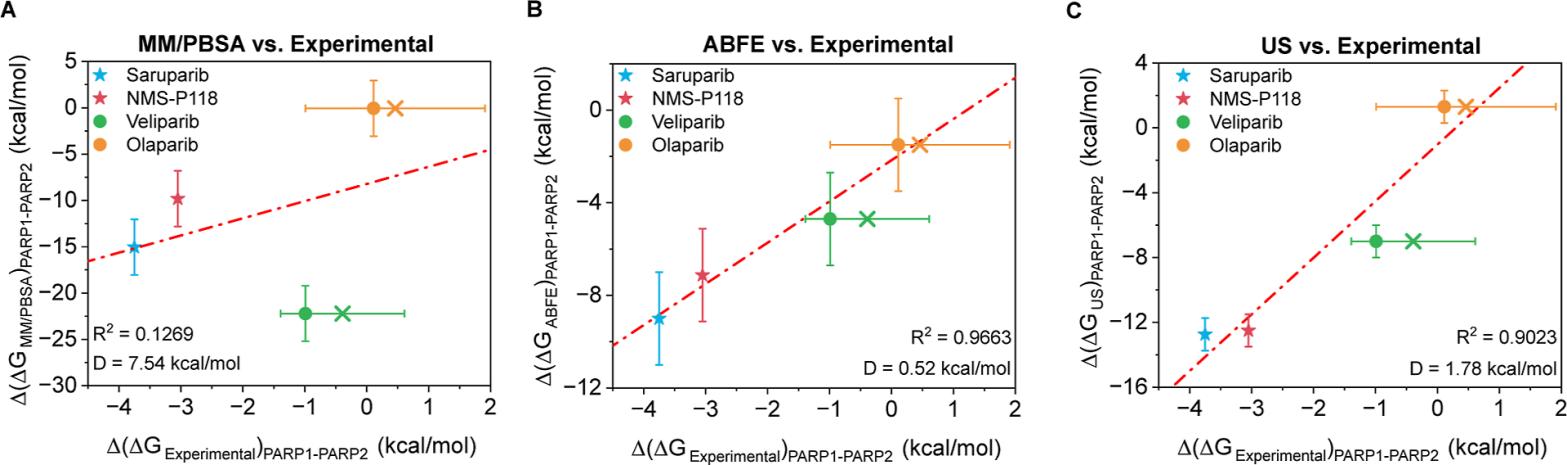
Comparison of the difference of binding free energy between PARP1 and PARP2 (Δ­(Δ*G*)_PARP1–PARP2_) for four inhibitors: saruparib (blue star), NMS-P118 (red star), veliparib (green circle), and olaparib (yellow circle) using different computational approaches. (A) Correlation between MM/PBSA and experimental results.^15,43–45^ (B) Correlation between ABFE and experimental results.^15,43–45^ (C) Correlation between US and experimental results.^15,43–45^ The crosses indicate the mean IC_50_ values extracted from the ChEMBL database, while filled circles depict the most cited IC_50_ value in literature. Error bars represent the standard deviation of the simulations, and the estimated experimental uncertainty from different reported IC_50_ values using eq 10. Specific inhibitors of PARP1 are plotted as stars and nonspecific inhibitors as circles. The red dashed line depicts the linear regression of the data shown.

A notorious improvement is obtained with ABFE, as shown in Figure 3B. Here, the correlation is remarkably strong, with *R* ^2^ = 0.9663, demonstrating that ABFE calculations in combination with the amber99sb-*disp*/OpenFF force fields nearly quantitatively capture all of the experimental variance across the ligand set. The deviation from the ideal linear fit decreases significantly to *D* = 0.52 kcal/mol, well within the typical statistical uncertainty associated with ABFE simulations. All four ligands fall close to the diagonal, and no significant outliers are observed, confirming that the force field accurately reproduces both the direction and magnitude of the experimental selectivity shifts.

Finally, the US comparison is shown in Figure 3C. The US-derived differences in binding free energy between PARP1 and PARP2 also correlate strongly with experiments, yielding *R* ^2^ = 0.9023 and a deviation of *D* = 1.78 kcal/mol. The magnitude of *D* lies close to the intrinsic uncertainty expected for US calculations (approximately 1 kcal/mol^28,77^ ). Importantly, the US approach correctly separates highly selective inhibitors such as saruparib and NMS-P118 from broadly active ones such as veliparib and olaparib, mirroring experimental trends. A key advantage of PMF-based approaches is that, although positional restraints are required to define the reaction coordinate, the resulting free energy profiles preserve a physical interpretation.^82^ Unlike ABFE calculations, in which absolute binding free energies are not directly meaningful unless the thermodynamic loop is closed and only differences between carefully constructed alchemical states can be interpreted, a PMF describes the free energy landscape along a physically defined reaction coordinate (Figure S1). This representation explicitly captures intermediate states, desolvation free energy barriers, and metastable configurations that arise during the ligand’s approach and accommodation within the binding pocket. Consequently, US calculations provide quantitative insight not only into the effective binding strength but also into the mechanism, accessibility of the binding site, and the energetic cost associated with ligand insertion and rearrangement through a given dissociation pathway. As demonstrated in our previous work,^82^ PMF profiles can yield quantitatively reliable relative binding free energies for closely related systems while simultaneously offering mechanistic information—such as desolvation free energy barriers^83^—that are inherently inaccessible through standard alchemical ABFE analyses based solely on Δ*G* differences.

It is also important to emphasize that the computational Δ*G* and Δ­(Δ*G*) values are not expected to match the experimental numbers quantitatively. Each method introduces intrinsic approximations that restrict the accessible conformational space of the protein–ligand complex. In particular, US simulations constrain the motion along a predefined reaction coordinate. These restraints reduce the accessible configurational entropy of the system and prevent exhaustive exploration of all relevant microstates, therefore limiting quantitative agreement with experiments beyond the intrinsic limitations of the employed force field. Even ABFE, despite its rigorous statistical foundation, samples only a subset of the full conformational ensemble owing to the finite simulation time. Consequently, the appropriate comparison between computational and experimental values should preferably be based on relative trends and selectivity patterns rather than on absolute binding free energies.

Taken together, the results of Figure 3 reveal a clear hierarchy of the predictive performance. MM/PBSA shows the weakest agreement with the experiment, exhibiting both the lowest correlation and the largest deviation values (eq 8). This reflects well-known limitations of end-point approaches, which rely on single-configuration enthalpic estimates and do not explicitly account for the many microstates and entropic contributions that determine the binding free energy. On the other hand, US and ABFE perform substantially better. US incorporates explicit solvent and partial configurational freedom along a reaction coordinate, and ABFE achieves the most complete sampling of conformations and intermolecular interactions accessible during ligand binding within the protein pocket. As a result, both display stronger linear correlations with the experimental selectivities and significantly smaller deviation values. Among them, ABFE yields the closest quantitative agreement, followed by US, which can be slightly affected by the positional restraints imposed during the reaction-coordinate sampling, as recently evidenced by us in refs 83 and 84 .

### Key Intermolecular Interactions Driving Ligand Selectivity in PARP1 Inhibition

To complement the free energy analysis presented above, we expand our data set beyond the four PARP inhibitors originally examined by including four additional clinically relevant compounds. The motivation for this extension is 2-fold. First, we incorporate a broader chemical diversity to assess how the trends in selectivity are determined by specific intermolecular contacts across the binding pocket. Second, this enlarged set enables a more reliable comparison between the two fastest computational approaches used to estimate binding affinity: MM/PBSA and ABFE. By analyzing the full panel of eight inhibitors with both methods, we aim to determine whether the additional compounds reinforce—or challenge—the selectivity patterns inferred from the initial data set.

In Figure 4, we summarize the comparison between the computed and experimental differences in binding free energy between PARP1 and PARP2 (Δ­(Δ*G*)_PARP1–PARP2_) for the full set of eight inhibitors. First, we report the MM/PBSA-derived selectivities (Figure 4A), while in Figure 4B, we show the corresponding ABFE predictions. The experimental IC_50_ values for the newly added inhibitors were taken as follows: rucaparib, 3.2 nM for PARP1 and 28.2 nM for PARP2;^46^ niraparib, 3.8 nM for PARP1 and 2.1 nM for PARP2;^47^ talazoparib, 2.59 nM for PARP1 and 0.89 nM for PARP2;^48^ and pamiparib, 1.3 nM for PARP1 and 0.9 nM for PARP2 ^49^ (see Table 1 for the experimental IC_50_ values used in this study, along with the chemical structures of the different PARP inhibitors). These values were subsequently converted into binding free energy differences (Δ*G*) (eq 9) for direct comparison with the computed results. When the four additional compounds are incorporated into the analysis, the overall performance of the two methods evolves in distinct ways.

**Figure 4:**
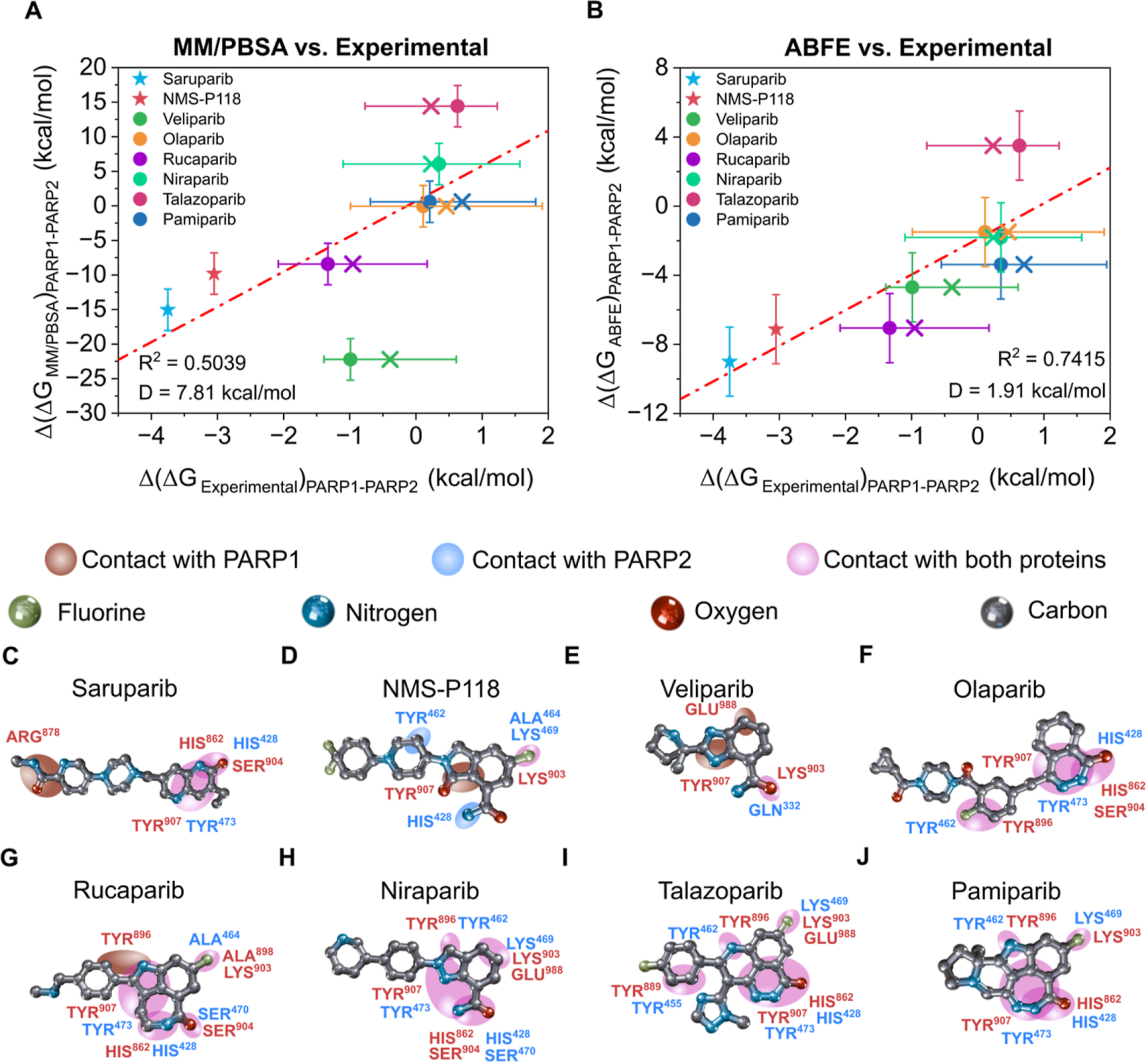
Comparison of binding free energy differences (ΔΔ*G*) between PARP1 and PARP2 for eight inhibitors: saruparib (blue star), NMS-P118 (red star), veliparib (green circle), olaparib (yellow circle), rucaparib (purple circle), niraparib (mint circle), talazoparib (maroon circle), and pamiparib (dark-blue circle) using different computational approaches. (A) Correlation between MM/PBSA and experimental results.^15,43–49^ (B) Correlation between ABFE and experimental results.^15,43–49^ Crosses indicate the mean IC_50_ values extracted from the ChEMBL database, while filled circles depict the most cited IC_50_ value from the literature for each ligand. Error bars represent the standard deviation of the simulations, and the estimated experimental uncertainty from different reported IC_50_ values using eq 10. Specific inhibitors of PARP1 are plotted as stars and nonspecific inhibitors as circles. Red dashed line depicts the linear regression of the data shown. Chemical structures of eight PARP inhibitors (C–J) annotated with their corresponding functional groups and regions establishing key contacts with the residues at the binding pockets in PARP1 (red labels) and PARP2 (blue labels). Panel C corresponds to saruparib, followed by NMS-P118 (D), veliparib (E), olaparib (F), rucaparib (G), niraparib (H), talazoparib (I), and pamiparib (J). Atom colors follow the scheme shown in the top legend: carbon (gray), nitrogen (blue), oxygen (red), and fluorine (green). Colored ellipses indicate protein–ligand contacts: brown marks regions contacting with PARP1, light blue marks regions contacting with PARP2, and purple denotes regions interacting with both proteins.

For MM/PBSA (Figure 4A), the agreement with experiment improves noticeably relative to the results obtained for the four initial inhibitors (Figure 3A). The coefficient of determination increases from *R* ^2^ = 0.2673 to *R* ^2^ = 0.5039, while the average deviation in binding energy difference remained roughly constant, *D* = 7.81 kcal/mol. This improvement in *R* ^2^ indicates that the additional data dilutes the strong influence of the large error previously introduced by veliparib, which has disproportionately affected the regression when only four ligands are considered. As a result, the MM/PBSA yields a more defined correlation once the chemical diversity of the data set is expanded. Moreover, if veliparib is excluded from the analysis, both statistical metrics improve substantially, with *R* ^2^ increasing to 0.7898 and the deviation decreasing to *D* = 4.3 kcal/mol. This reduced deviation approaches the intrinsic error typically associated with MM/PBSA calculations,^75^ highlighting that this method performs reasonably well for the remaining ligands when this potential outlier is removed. In contrast, the ABFE calculations (Figure 4B) show the opposite trend. Although it remains the more accurate method overall, its performance slightly deteriorates compared with the initial analysis (Figure 3B), giving *R* ^2^ = 0.7415 and *D* = 1.91 kcal/mol. Nevertheless, the advantage of using ABFE is still notable since its average deviation in binding free energy difference for the whole set of inhibitors remains over four times lower than that obtained through MM/PBSA calculations for the eight inhibitors.

MM/PBSA calculations in this work were performed by using the standard single-trajectory protocol, which remains one of the most commonly employed implementations in computational binding free energy studies. Several methodological extensions—including multitrajectory schemes, ensemble-based strategies, and multiscale approaches—have been recently proposed to improve the robustness and predictive performance of MM/PBSA calculations.^56^ These more advanced variants were not explored in the present study, as our objective was to compare standard, widely used implementations of the different methods within a consistent methodological framework. Accordingly, the results reported here should be interpreted in the context of the specific MM/PBSA protocol employed.

To further test the robustness of our computational protocol, we perform additional ABFE calculations for niraparib bound to two distant PARP-family members: PARP15 and TNK1, whose IC_50_ values (29,200 nM^85^ and 40,000 nM,^86^ respectively) lie far outside the range observed for PARP1 and PARP2. As shown in Figure S2 of the Supporting Information, these ABFE simulations also correctly reproduce the expected qualitative behavior, yielding markedly weaker binding for PARP15 and TNK1 relative to that for PARP1 and PARP2. This again confirms that our methodology is capable of resolving large selectivity differences across more divergent targets. By contrast, the selectivity between PARP1 and PARP2 represents an exceptionally demanding test case. The two catalytic domains are highly homologous, and the free energy differences that distinguish selective from nonselective inhibitors are rather small—often within the subkcal/mol regime where both experimental uncertainty and the intrinsic statistical noise of alchemical calculations become comparable to the signal itself. In this context, our ABFE calculations performed here push the method to the edge of its practical resolution. The fact that ABFE nonetheless recovers coherent and chemically meaningful trends across both the initial and expanded inhibitor sets underscores the robustness of the a99SB-*disp*/OpenFF all-atom force field, as well as supporting the conclusion that the observed selectivity patterns are not artifacts of methodological choices since they are also consistent with previous US calculations (Figure 2C).

It is also worth noting that Figure S3A,C in the Supporting Information presents the results of the MM/PBSA calculations without the entropic term (S3A), along with the corresponding MM/GBSA estimates without and with entropy (S3B and S3C, respectively). These data clearly show that omitting the entropic contribution systematically worsens the predictive performance in both approaches. More importantly, the comparison between MM/PBSA and MM/GBSA highlights a markedly superior performance of MM/PBSA for this class of systems: the PB-based solvation model yields more stable and consistent trends, whereas the GB variant exhibited a stronger sensitivity to both the dielectric environment and the conformational heterogeneity of the complexes^87^ (in Figure S4 of the Supporting Information are shown the charged amino acids of both protein active sites). This observation helps rationalize why, for structurally complex and highly charged binding pockets such as those examined here, MM/PBSA provides a framework that is more reliable than MM/GBSA for estimating relative binding free energies.

By extending the MD trajectories from ABFE calculations to 0.2 μ*s*, a detailed molecular analysis of residue-level contacts is also performed to explore whether structural and intermolecular interacting patterns can explain the selectivity differences observed between PARP1 and PARP2. The contact analysis is based on distances between heavy atoms of the ligand and the centers of mass of amino acid side chains (see Section VI for methodological details). While free energy calculations quantify the relative binding affinities, they do not explicitly reveal the microscopic molecular determinants responsible for protein selectivity. For this reason, we examine the most frequent intermolecular contacts formed by each inhibitor within the catalytic binding site. The goal of this analysis is to identify recurring structural motifs—such as electrostatic interactions, conserved aromatic stacking, or differences in hydrogen-bonding networks—that might serve as key markers of molecular preference. By comparing the contact maps across selective and nonselective inhibitors, we sought to determine whether a consistent structural pattern, which correlates with experimentally known selectivity, may emerge. For clarity, the main interaction patterns are summarized schematically in Figure 4, while a detailed residue-level contact table reporting the specific protein residues and ligand atoms involved in these interactions is provided in Tables S1 and S2 of the Supporting Information.

For specific inhibitors, such as saruparib in Figure 4C, we reveal a binding mode in which key dominant contacts—HIS^862^ and the aromatic stacking with TYR^907^ (corresponding to residues HIS^428^ and TYR^473^ in PARP2)—are partially shared between PARP1 and PARP2. The only two contacts that saruparib exclusively establishes in PARP1 are with ARG^878^ and SER^904^, which do not appear in PARP2 binding. Given that both proteins present an overall similar interaction pattern, the presence of these additional PARP1-specific interactions seems to be crucial for the experimentally observed selectivity for PARP1. In Figure 4D, NMS-P118 shows a different scenario. While both NMS-P118 and saruparib shared several stabilizing contacts—most notably TYR^907^ and LYS^903^—NMS-P118 also forms a PARP1-exclusive interaction with TYR^907^ and PARP2 presents its own set of moderate stabilizing contacts with NMS-P118, including TYR^462^, HIS^428^, and ALA^464^ among others. This pattern suggests that cross-interactions with TYR^907^ and LYS^903^ in PARP1 might be critical for NMS-P118 to behave as a selective inhibitor of PARP1. Nevertheless, beyond the leading interactions with these two protein residues in PARP1, our data also suggests that selectivity likely arises from multiple, less dominant cooperative intermolecular contacts within each protein. The combination and synergy of these contacts further enhance nonshared interactions that differ significantly between PARP1 and PARP2 (Figures S5–S8).

In contrast, poorly specific inhibitors, such as veliparib (Figure 4E), show an interaction pattern with both proteins that shares key contacts between heavy atoms of the ligand and protein residues, including LYS^903^ in PARP1, or GLN^332^ in PARP2. Beyond these shared anchoring interactions, the remaining contacts are predominantly observed in PARP1, particularly with GLU^988^ and TYR^907^. This suggests that although veliparib engages both PARP1/PARP2 binding pockets through a similar recognition mechanism, the larger number of stabilizing interactions formed specifically with PARP1 may explain why both MM/PBSA and US predict a slight preference toward PARP1. Olaparib (Figure 4F) represents the opposite scenario. All major contacts are shared between the two proteins involving HIS^862^, TYR^896^, and TYR^907^ in PARP1, which correspond to HIS^428^, TYR^462^, and TYR^473^ in PARP2. The complete overlap in residue-level interactions further supported our ABFE, US, and MM/PBSA calculations, indicating that olaparib adopts essentially identical binding modes in both protein catalytic pockets. Such structural equivalence is fully consistent with the experimental observation that olaparib shows similar selectivity for both PARP1 and PARP2.

For rucaparib (Figure 4G), multiple intermolecular contacts are shared between PARP1 and PARP2, including TYR^907^, HIS^862^, SER^904^, and ALA^898^ in PARP1, as well as TYR^473^, HIS^428^, SER^470^, and ALA^898^ in PARP2. Notably, TYR^896^ and SER^904^ are observed exclusively in PARP1. The presence of these PARP1-specific interactions suggests that rucaparib may exhibit partial selectivity toward PARP1—as experimentally shown in Figure 4A—despite also forming multiple stabilizing contacts with PARP2. In contrast, niraparib (Figure 4H), talazoparib (Figure 4I), and pamiparib (Figure 4J) predominantly form contacts that are shared across both proteins. Key residues involved in these homologous interactions include TYR^896^, TYR^907^, HIS^862^, and LYS^903^, which are conserved in both PARP1 and PARP2 catalytic binding sites. The extensive overlap in the intermolecular contact patterns indicates that these inhibitors utilize a similar binding mechanism in both proteins and consequently cannot display significant selectivity. Overall, these structural observations reveal how contact connectivity controls ligand selectivity. Furthermore, our analysis provides valuable mechanistic and molecular insights into the chief residues that stabilize each ligand in both proteins (an energetic decomposition of the residues that contribute most strongly to ligand stabilization in each protein is shown in Figures S5–S8), helping to elucidate potential chemical modifications across the ligand chemical space to enhance potency and specificity. Thus, structural contact analysis remains an informative qualitative tool for interpreting free energy trends and guiding the development and optimization of next-generation oncologic drugs.

## Conclusions

In this work, we systematically evaluate the ability of three computational methods—MM/PBSA, ABFE, and US—to predict ligand inhibitor selectivity in two protein paralogues, PARP1 and PARP2, used as therapeutic targets in precision oncology. Our multimethod analysis reveals a clear progression in both methodological rigor and predictive performance. MM/PBSA, which relies on static energy-based estimations with an implicit solvent, provides a rapid and qualitative view of relative selectivity. However, its predictions do not consider molecular conformational rearrangements, solvent-mediated interactions, or real entropic contributions. Nevertheless, despite these aggressive approximations, MM/PBSA captured general trends among PARP1/PARP2 inhibitors (Figure 4A), suggesting that it can serve as a useful initial screening tool for ligand selectivity.

ABFE calculations using the all-atom a99SB-*disp* and OpenFF force fields, in contrast, achieve a much higher predictive accuracy. By explicitly sampling protein–ligand interactions and solvent dynamics, ABFE calculations using this all-atom force field reproduce experimental selectivity patterns with remarkable fidelity. Across both the initial and expanded inhibitor sets, ABFE consistently captures the trend and magnitude of binding free energy differences between PARP1 and PARP2, demonstrating that phase-space sampling is essential for resolving the subtle energetic distinctions that dictate selectivity between highly homologous proteins (Figures 3B and 4B). Finally, all-atom US calculations also using the a99SB-*disp* and OpenFF force fields show strong agreement with experimental data (Figure 3C), correctly differentiating selective from nonselective inhibitors. Nevertheless, this method—which is the most computationally expensive of the three studied techniques (Figure 2A)—exhibits slight smoothing of energetic differences due to the required constraints of choosing a predefined dissociation pathway, which can partially limit discrimination among highly selective ligands.

Furthermore, our structural analyses elucidate the contact connectivity that controls ligand selectivity in PARP1. We reveal that while highly selective inhibitors such as saruparib and NMS-P118 form few protein-specific contacts—such as ARG^878^ and TYR^907^ by saruparib, or LYS^903^ and TYR^907^ by NMS-P118—most clinically used PARP inhibitors interact extensively with residues conserved across both PARP1 and PARP2 (Figure 4C–J). These findings highlight why computer simulations, including contact connectivity analyses and free energy calculations, are vital to rationalize and propose new chemical modifications to enhance ligand specificity and potency. Taken together, our work highlights the critical role of rigorous calculations in computational drug design and establishes a robust framework to guide the development of selective ligands for next-generation cancer therapeutics.

## Supplementary Material





## Data Availability

We provide the relevant data in the repository (https://github.com/Reshiiiii/PARP_MultiMethod_Data_Scripts) to facilitate reproducibility of our results. In the repository, we also give the necessary code to run the simulations and accessible instructions to obtain our results.

## Notes

Detailed computational methods including MM/PB­(GB)­SA calculations, system preparation, and all-atom MD simulations; ABFE alchemical free energy calculations with MBAR analysis; US simulations with WHAM analysis; contact map calculation protocol; atomistic PMF dissociation profiles for multiple ligands with PARP1 and PARP2; comparison between experimental and ABFE-predicted selectivity of PARP1 versus off-targets (TNK1, PARP15); binding free-energy differences (ΔΔ*G*) between PARP1 and PARP2 for eight inhibitors using MM/PBSA, MM/GBSA, and experimental data; schematic representation of charged amino acids in the active sites of PARP1 and PARP2, showing positively and negatively charged residues; total energy decomposition of different ligands in the active-site pockets of PARP1 and PARP2; overlap matrices for alchemical ABFE of saruparib in PARP1 and PARP2; and residue–atom contacts identified for the ligands in complex with PARP1 and PARP2 (PDF)
Force field data and scripts (ZIP)
