# Supplementary material for: Comparative Assessment of Free Energy Computational Methods for Revealing the Interactions Driving PARP1 Selective Inhibition
Source: J Chem Inf Model. 2026 Apr 18;66(9):5315–30. doi: 10.1021/acs.jcim.6c00083 (PMC13169362; doi:10.1021/acs.jcim.6c00083)
Supplement: Supplementary file 2 [file ci6c00083_si_002.zip › Data_FF/Force_Field.zip!/Force_Field/a99SBdisp.ff/forcefield.doc]

a99SBdisp

********************************************************************

PLEASE CITE:

Developing a molecular dynamics force field for both folded and disordered protein states

P Robustelli, S Piana, DE Shaw

Proceedings of the National Academy of Sciences 115 (21), E4758-E4766

********************************************************************
